# Retraction: Pan, M. et al. Enhanced Adsorption of Zn(II) onto Graphene Oxides Investigated Using Batch and Modeling Techniques. *Nanomaterials* 2018, *8*, 806

**DOI:** 10.3390/nano10061180

**Published:** 2020-06-17

**Authors:** Min Pan, Guangxue Wu, Chang Liu, Xinxin Lin, Xiaoming Huang

**Affiliations:** 1 Fujian Engineering and Research Center of Rural Sewage Treatment and Water Safety, School of Environmental Science and Engineering, Xiamen University of Technology, Xiamen 361024, China; lin.xinxin@outlook.com; 2 Graduate School at Shenzhen, Tsinghua University, Shenzhen 518055, China; wu.guangxue@sz.tsinghua.edu.cn; 3 College of Environmental Science and Engineering, Anhui Normal University, Wuhu 241002, China; lc2014@ahnu.edu.cn

This article (ref. 1) has been retracted at the request of the authors, as Figure 1A is similar with Figure 1B of the article published by Sun et al. in 2015 [1]. Moreover, a section of Figure 1B in (ref. 1) is similar with a section of Figure 1B of the article published by Sun et al. in 2015 [2].

The decision to retract has been taken in agreement with the authors and approved by the Editor-in-Chief.

The *Nanomaterials* Editorial Office and authors apologize to the readers of *Nanomaterials* for any inconvenience caused. To ensure the addition of only high-quality scientific work to the field of scholarly publication, this paper (ref. 1) is retracted and shall be marked accordingly. MDPI is a member of the Committee on Publication Ethics (COPE) and takes the responsibility to enforce strict ethical policies and standards very seriously.
